# The fractional nonlinear ${\mathcal{PT}}$ dimer

**DOI:** 10.1038/s41598-021-89484-x

**Published:** 2021-05-11

**Authors:** Mario I. Molina

**Affiliations:** Departamento de Física, Facultad de Ciencias, Universidad de Chile, Casilla 653, Santiago, Chile

**Keywords:** Statistical physics, thermodynamics and nonlinear dynamics, Mathematics and computing, Physics

## Abstract

We examine a fractional discrete nonlinear Schrodinger dimer, where the usual first-order derivative in the time evolution is replaced by a non integer-order derivative. The dimer is nonlinear (Kerr) and ${\mathcal{{PT}}}$-symmetric, and for localized initial conditions we examine the exchange dynamics between both sites. By means of the Laplace transformation technique, the linear ${{\mathcal{{PT}}}}$ dimer is solved in closed form in terms of Mittag–Leffler functions, while for the nonlinear regime, we resort to numerical computations using the direct explicit Grunwald algorithm. In general, we see that the main effect of the fractional derivative is to produce a monotonically decreasing time envelope for the amplitude of the oscillatory exchange. In the presence of ${\mathcal{{PT}}}$ symmetry, the oscillations experience some amplification for gain/loss values below some threshold, while beyond threshold, the amplitudes of both sites grow unbounded. The presence of nonlinearity can arrest the unbounded growth and lead to a selftrapped state. The trapped fraction decreases as the nonlinearity is increased past a critical value, in marked contrast with the standard (non-fractional) case.

## Introduction

The topic of fractional calculus has experienced a rekindled interest in recent times. Essentially, it extends the notion of a derivative or an integral of integer order, to one of a fractional order, $(d^{n}/dx^{n})\rightarrow (d^{\alpha }/d x^{\alpha })$ for real $\alpha$. The subject has a long history, dating back to letters exchanged between Leibnitz and L’Hopital, and later contributions by Euler, Laplace, Riemann, Liouville, and Caputo to name some^1–5^. The starting point was the computation of $d^{\alpha } x^{k}/d x^{\alpha }$, where $\alpha$ is a non-integer number:

1 $$\begin{aligned} {d^{n} x^k\over {d x^{n}}}= {\Gamma (k+1)\over {\Gamma (k-n+1)}} x^{k-n} \rightarrow {d^{\alpha } x^k\over {d x^{\alpha }}} = {\Gamma (k+1)\over {\Gamma (k-\alpha +1)}} x^{k-\alpha }. \end{aligned}$$

For instance, $(d^{1/2}/d x^{1/2}) x = (2/\sqrt{\pi }) \sqrt{x}$, and $d x/d x = (d^{1/2}/d x^{1/2}) (d^{1/2}/d x^{1/2}) x = (2\sqrt{\pi })(\Gamma (3)/\Gamma (1)) x^{0} = 1$, as expected. From Eq. (1) the fractional derivative of an analytic function $f(x)=\sum _{k} a_{k} x^{k}$ can be computed by deriving term by term. This basic procedure is not exempt from ambiguities. For instance, $(d^{\alpha }/d x^{\alpha })\ 1=(d^{\alpha } x^{0}/d x^{\alpha })=(1/\Gamma (1-\alpha )) x^{-\alpha }\ne 0$, according to Eq. (1). However, one could have also taken $(d^{\alpha -1}/d x^{\alpha -1})(d/dx)\ 1=0$. For the case of a fractional integral, a more rigorous starting point is Cauchy’s formula for the integral of a function. From the definition

2 $$\begin{aligned} I_{x}^{1} f(x) = \int _{0}^{x} f(s) d s, \end{aligned}$$

we apply the Laplace transform ${{\mathcal{L}}}$ to both sides of Eq. (2)

3 $$\begin{aligned} {{{\mathcal{L}}}}\ \big \{I_{x}^{1}\ f(x)\big \}= (1/s)\ {{{\mathcal{L}}}}\{f(x)\}. \end{aligned}$$

After *n* integrations, one obtains

4 $$\begin{aligned} {{{\mathcal{L}}}}\ \big \{I_{x}^{n}\ f(x)\big \}= (1/s^{n})\ {{{\mathcal{L}}}}\{f(x)\}. \end{aligned}$$

Extension to fractional $\alpha$ is direct:

5 $$\begin{aligned} {{{\mathcal{L}}}}\ \big \{I_{x}^{\alpha }\ f(x)\big \}= (1/s^{\alpha })\ {{{\mathcal{L}}}}\{f(x)\}. \end{aligned}$$

After noting that the RHS of Eq. (5) is the product of two Laplace transforms we have, after using the convolution theorem

6 $$\begin{aligned} I_{x}^{\alpha }\ f(x) = {1\over {\Gamma (\alpha )}} \int _{0}^{x} {f(s)\over {(x-s)^{1-\alpha }}}\ ds. \end{aligned}$$

From this definition, it is possible to define the fractional derivative of a function *f*(*x*) as

7 $$\begin{aligned} {d^{\alpha } f(x)\over {d x^{\alpha }}} = \left( {d^m \over {d x^m}}\right) \ I_{x}^{m-\alpha }\ f(x) = {d^{m}\over {d x^m}} \left[ {1\over {\Gamma (m-\alpha )}} \int _{0}^{x} (x-s)^{m-\alpha -1} f(s)\ ds\ \right] , \end{aligned}$$

where $m-1<\alpha <m$. Equation (7) is known as the Riemann–Liouville form. An alternative, closely related form, is the Caputo formula^5^:

8 $$\begin{aligned} {d^{\alpha } f(x)\over {d x^{\alpha }}} = I_{x}^{m-\alpha }\left( {d^{m}\over {d x^m}}\right) f(x) = {1\over {\Gamma (m-\alpha )}} \int _{0}^{x}(x-s)^{m-\alpha -1} f^{(m)}(s) ds, \end{aligned}$$

which has some advantages over the Riemann–Liouvuille form for differential equations with initial values. The various technical matters that arise in fractional calculus have prompted a whole line of research that has extended to current times. Long regarded as a mathematical curiosity, it has now regained interest due to its potential applications to complex problems in several fields: fluid mechanics^6,7^, fractional kinetics and anomalous diffusion^8–10^, strange kinetics^11^, fractional quantum mechanics^12,13^, Levy processes in quantum mechanics^14^, plasmas^15^, electrical propagation in cardiac tissue^16^ and biological invasions^17^. In general, fractional calculus constitutes a natural formalism for the description of memory and non-locality effects found in various complex systems. Experimental realizations are not straightforward given the nonlocal character of the coupling, however some optical setups have been suggested that could measure the effect of fractionality on new beam solutions and new optical devices^18,19^.

On the other hand, when dealing with effectively discrete, interacting units, as one encounters in atomic physics (interacting atoms), or in optics ( coupled optical fibers), it is common to deal with discrete versions of the continuum Schrödinger equation, or the paraxial wave equation. The effective discreteness comes from expanding the solution sought in terms of (continuous) modes that can be labelled unambiguously. The simplest of such examples is the bonding, anti-bonding electronic mode that one finds for a two-sites (dimer) molecule after diagonalizing the two-site Schrödinger equation in the tight-binding approach. Something similar happens in optics, where the paraxial equation is formally equivalent to the Schrödnger equation. In that case, for two optical waveguides, the total electric field is expanded in terms of the electromagnetic modes in each guide which interact through the evanescent field between the two guides giving rise to a transversal dynamics for the optical power. The procedure can be extended to *N* interacting units, either atoms or waveguides, where the relevant dynamics is given by a discretized version of the Schrödinger equation for *N* units^20,21^. Of course, at the end one has to collect all the discrete amplitudes and multiply them by the corresponding continuous mode profiles and superpose them, to obtain the final field. The simplest case $N=2$ is termed a dimer and oftentimes constitute a basic starting point when studying an interacting, discrete system. Ensembles of interacting dimers have been studied before in classical and quantum statistics^22–24^, and more recently, they have been considered in model of correlated disorder^25^ and in magnetic metamaterial modeling^26^.

**Figure 1:**
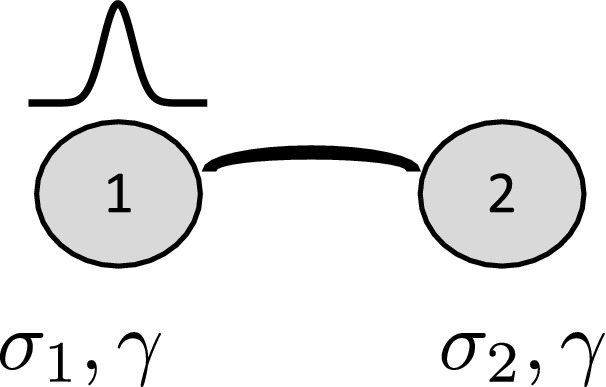
Nonlinear anisotropic fractional dimer where the excitation is on site 1 initially ($t=0$). In the ${\mathcal{{PT}}}$ case, $\sigma _{1}=-\sigma _{2}=i \sigma$.

In this work we consider the discrete Schrödinger equation for a dimer system, where the standard time derivative is replaced by a fractional one. The dimer considered is rather general and contains fractionality, $\mathcal{PT}$ symmetry and nonlinearity (Fig. 1). Our main interest is in ascertaining the effect of the fractional derivative on the excitation exchange between the sites, its stability and selftrapping behavior, for several cases of interest.

## The fractional dimer

The standard dimer has the form

9 $$\begin{aligned} i {d C_{1}(t)\over {d t^{\alpha }}} + \epsilon _{1} C_{1}(t) + V C_{2}(t) + \chi |C_{1}(t)|^2 C_{1}(t)= & {} 0\nonumber \\ i {d C_{2}(t)\over {d t^{\alpha }}} + \epsilon _{2} C_{2}(t) + V C_{1}(t) + \chi |C_{2}(t)|^2 C_{2}(t)= & {} 0\ \ \ \ \end{aligned}$$

where quantities $C_{1,2}$ are probability amplitudes in a quantum context, or electric field amplitudes, in an optical setting. Parameter *V* is the coupling term and $\chi$ is the nonlinearity parameter. Before going into the fractional version of this equation, it is convenient to rewrite Eq. (9) in a dimensionless form^27^. We define $\phi _{1,2} = C_{1,2}/C_{0}$ as the dimensionless amplitudes, where $C_{0}$ is a characteristic amplitude, like $(1/2)(C_{1}(0)+C_{2}(0))$. Also, we define $z=V t$ as the dimensionless time, $\sigma _{1,2}=\epsilon _{1,2}/V$ as the dimensionless site energies, and $\gamma =(\chi /V) C_{0}^2$ as the dimensionless nonlinearity. In terms of the new variables, we have

10 $$\begin{aligned} i {d \phi _{1}(z)\over {d z}} + \sigma _{1} \phi _{1}(z) + \phi _{2}(z) + \gamma |\phi _{1}(z)|^2 \phi _{1}(z)= & {} 0\nonumber \\ i {d \phi _{2}(z)\over {d z}} + \sigma _{2} \phi _{2}(z) + \phi _{1}(z) + \gamma |\phi _{2}(z)|^2 \phi _{2}(z)= & {} 0\ \ \ \ \end{aligned}$$

We now go into the fractional version of the model by adopting a fractional derivative, $(d/d z) \rightarrow (d^{\alpha }/d z^{\alpha })$ in Eq. (10):

11 $$\begin{aligned} i {d^{\alpha } \phi _{1}(z)\over {d z^{\alpha }}} + \sigma _{1} \phi _{1}(z) + \phi _{2}(z) + \gamma |\phi _{1}(z)|^2 \phi _{1}(z)= & {} 0\nonumber \\ i {d^{\alpha } \phi _{2}(z)\over {d z^{\alpha }}} + \sigma _{2} \phi _{2}(z) + \phi _{1}(z) + \gamma |\phi _{2}(z)|^2 \phi _{2}(z)= & {} 0\ \ \ \ \end{aligned}$$

Equation (11) needs two arbitrary constants. This can be seen, for instance, by means of elementary manipulations and using the composition property of the fractional derivative, to re-cast (11) in the linear limit ($\gamma =0$) as a decoupled system

12 $$\begin{aligned} (d^{2\alpha }/d z^{2\alpha }) \phi _1 - i (\sigma _1+\sigma _2) (d^{\alpha }/d z^{\alpha }) \phi _1 + (1-\sigma _{1}\sigma _{2}) \phi _{1}= & {} 0\nonumber \\ (d^{2\alpha }/d z^{2\alpha }) \phi _2 - i (\sigma _1+\sigma _2) (d^{\alpha }/d z^{\alpha }) \phi _2 + (1-\sigma _{1}\sigma _{2}) \phi _{2}= & {} 0 \end{aligned}$$

According to the general theory of fractional calculus^3^, each of Eq. (12) needs just a single arbitrary constant, for $0<\alpha <1$. Thus, for our coupled system (11), just two arbitrary constants are required.

Let us first consider the case of a general linear ($\gamma =0$) dimer, and assume $\phi _{1}(0)=1, \phi _{2}(0)=0$. We will solve this case in closed form by the use of Laplace transforms: For $0<\alpha <1$, the Laplace transform of the Caputo fractional derivative of order $\alpha$ is given by

13 $$\begin{aligned} {{{\mathcal{L}}}}\{f^{(\alpha)} (z)\} = s^{\alpha } {{{\mathcal{L}}}}\{f(z)\} - s^{\alpha -1} f(0^{+}). \end{aligned}$$

After applying the Laplace transform ${{\mathcal{L}}}$ to both sides of Eq. (11) we have

14 $$\begin{aligned} i (s^{\alpha } {{{\mathcal{L}}}}(\phi _{1}) - s^{\alpha -1}) + \sigma _{1} {{{\mathcal{L}}}}(\phi _{1})+ {{{\mathcal{L}}}}(\phi _{2})= & {} 0\nonumber \\ i\ s^{\alpha } {{{\mathcal{L}}}}(\phi _{2}) + \sigma _{2} {{{\mathcal{L}}}}(\phi _{2})+ {{{\mathcal{L}}}}(\phi _{1})= & {} 0. \end{aligned}$$

Solving for ${{{\mathcal{L}}}}(\phi _{1})$ and ${{{\mathcal{L}}}}(\phi _{2})$ gives:

15 $$\begin{aligned} {{{\mathcal{L}}}}(\phi _{1}) = {- i (\sigma _{2} + i s^{\alpha }) s^{\alpha -1}\over {s^{2 \alpha } - i (\sigma _{1}+\sigma _{2})s^{\alpha } + V^2-\sigma _{1} \sigma _{2}}} \end{aligned}$$

and

16 $$\begin{aligned} {{{\mathcal{L}}}}(\phi _{2}) = {i\ s^{\alpha -1}\over {s^{2 \alpha } - i (\sigma _{1} + \sigma _{2}) s^{\alpha } + 1-\sigma _{1} \sigma _{2}}}. \end{aligned}$$

**Figure 2:**
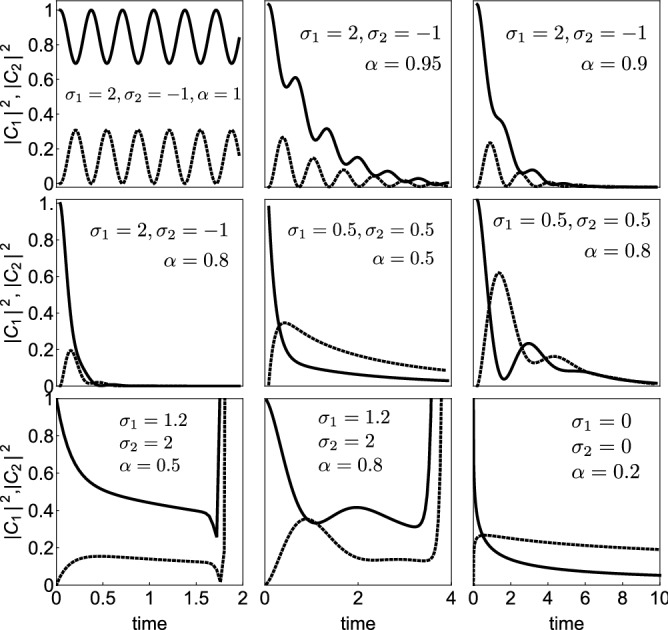
Dimer amplitudes for the linear case ($\gamma =0$) and several site energy parameters $\sigma _{1}, \sigma _{2}$ and different fractional derivative orders $\alpha$. Solid(dashed) line denotes $|\phi _{1}|^2$ $(|\phi _{2}|^2)$.

Using the inverse Laplace formula^28^

17 $$\begin{aligned}&{{{\mathcal{L}}}}^{-1}\left\{ {s^{\rho -1}\over {s^{\alpha }+a s^\beta +b}}\right\} = z^{\alpha -\rho }\sum _{r=0}^{\infty } (-a)^r z^{(\alpha -\beta )r} E_{\alpha ,\alpha +(\alpha -\beta )r-\rho +1}^{r+1} (-b z^{\alpha }),\ \ \ \ \end{aligned}$$

we obtain $\phi _{1}(z)$ and $\phi _{2}(z)$ in closed form:

18 $$\begin{aligned}&\phi _{1}(z) = \sum _{r=0}^{\infty } (i (\sigma _{1}+\sigma _{2}))^{r}\ z^{\alpha r} E_{2\alpha ,\alpha r +1}^{r+1} \big ((\sigma _{1}\sigma _{2}-1)z^{2 \alpha }\big )\nonumber \\&-i \sigma _{2} z^{\alpha }\sum _{r=0}^{\infty } (i (\sigma _{1}+\sigma _{2}))^r z^{\alpha r} E_{2\alpha ,\alpha (1+r)+1}^{r+1}\big ((\sigma _{1}\sigma _{2}-1) z^{2\alpha }\big )\ \ \ \ \ \ \ \ \ \end{aligned}$$

19 $$\begin{aligned}&\phi _{2}(z)=i z^{\alpha } \sum _{r=0}^{\infty } (i (\sigma _{1}+\sigma _{2}))^r z^{\alpha r} E_{2\alpha ,\alpha (1+r)+1}^{r+1} ((\sigma _{1}\sigma _{2}-1) z^{2\alpha }) \end{aligned}$$

where $E_{\alpha ,\beta }^{\delta }(z)$ is defined as

20 $$\begin{aligned} E_{\alpha ,\beta }^{\delta }(z) = \sum _{k=0}^{\infty } {(\delta )_{k}\ z^{k}\over {k!\ \Gamma (\alpha k + \beta )}} \end{aligned}$$

where $(\delta )_{n} = \Gamma (\delta +n)/\Gamma (\delta )$, and $\alpha , \beta , \delta \in {\mathbb {C}}$, ${\text{Re }}(\alpha )>0, {\text{ Re }}(\beta )>0, z\in {\mathbb {C}}$. Figure 2 shows examples of the time evolution of the square of the dimer amplitudes, for several site energy parameters, and fractional derivative orders. In general we observe that, as soon as $\alpha$ differs from unity, the dynamics is either bounded or unbounded, depending on the values of the site energy parameters. For the bounded cases, there is some oscillation initially, with a decreasing envelope towards zero.

### The linear ${\mathcal{{PT}}}$ dimer

A particularly interesting case of Eq. (9), is the fractional ${\mathcal{{PT}}}$-symmetric dimer. For systems that are invariant under the combined operations of parity (${{{\mathcal{P}}}}$) and time reversal (${{{\mathcal{T}}}}$), it was shown that they display a real eigenvalue spectrum, even though the underlying Hamiltonian is not hermitian^29,30^. In these systems there is a balance between gain and loss, leading to a bounded dynamics. However, as the gain/loss parameter exceeds a certain value the system undergoes a spontaneous symmetry breaking, where two or more eigenvalues become complex. At that point, the system loses its balance and its dynamics becomes unbounded. According to the general theory, for our system to be ${\mathcal{{PT}}}$ symmetric, the real part of the site energies in Eq. (9) must be even in space while the imaginary part must be odd: ${\text{Re }}(\sigma _{1})={\text{Re }}(\sigma _{2})$ and ${\text{Im }}(\sigma _{1})=-{\text{ Im }}(\sigma _{2})$. For simplicity we take the real parts of $\sigma _{1}$, $\sigma _{2}$ as zero and thus, $\sigma _{1}=-\sigma _{2}\equiv i\ \sigma$, where $\sigma$ is the gain/loss parameter. Recent work on the classical and quantum version of the linear ${\mathcal{{PT}}}$ dimer aim at ascertaining its potential for future light transport in optical circuits^31^.

Here, we explore the effect of using a fractional-order derivative for the ${\mathcal{{PT}}}$ dimer. The equations have the form

21 $$\begin{aligned} i {d^{\alpha } \phi _{1}(z)\over {d z^{\alpha }}} + i \sigma \phi _{1}(z) + \phi _{2}(z)= & {} 0\nonumber \\ i {d^{\alpha } \phi _{2}(z)\over {d z^{\alpha }}} - i \sigma \phi _{2}(z) + \phi _{1}(z)= & {} 0\ \ \ \ \end{aligned}$$

whose exact solutions can be extracted from the general solution, Eqs. (18) and (19) as

22 $$\begin{aligned} \phi _{1}(z)= & {} -\sigma z^{\alpha } E_{2 \alpha ,\alpha +1}\big ((\sigma ^2-1)z^{2 \alpha }\big ) + E_{2 \alpha ,1}\big ((\sigma ^2-1)z^{2 \alpha }\big )\nonumber \\ \phi _{2}(z) = & {} i z^{\alpha } E_{2 \alpha ,\alpha +1}\big ((\sigma ^2-1) z^{2 \alpha }\big ), \end{aligned}$$

where $E_{\alpha ,\beta }(z)=E_{\alpha ,\beta }^{1}(z)$ is known as the generalized Mittag–Leffler function

23 $$\begin{aligned} E_{\alpha , \beta }(z) = \sum _{k} {z^{k}\over {\Gamma (\alpha k + \beta )}}. \end{aligned}$$

The function $E_{\alpha , \beta }(z)$ is the natural extension of the exponential function and plays the same rol for fractional differential equations, as the exponential function does for the standard integer differential equations. The initial conditions used are inspired from optics where the dimer stands for a system of two coupled waveguides, where optical power is placed on one guide (“guide 1”) only, and the exchange dynamics is studied, in the presence of gain/loss and/or nonlinearity. Of course, more general initial conditions can be treated using this same Laplace formalism, or by superposing solutions with simple initial conditions in a judicious manner.

Two interesting limiting cases can be extracted from the general solution. The first one is the limit $\alpha \rightarrow 0$. In this case,

24 $$\begin{aligned} \phi _{1}(z)= & {} (1-\sigma )\ E_{0,1}(\sigma ^2-1)={1-\sigma \over {2-\sigma ^2}}\nonumber \\ \phi _{2}(z)= & {} i\ E_{0,1} (\sigma ^2-1) = {i\over {2-\sigma ^2}}, \end{aligned}$$

which could be interpreted as a linear selftrapping. In the absence of gain/loss, $|\phi _{1}|^2=|\phi _{2}|^2=1/4$. Further increase in $\sigma$ decreases the amplitudes and, when $\sigma \rightarrow \infty$, $|\phi _{1}|^2\rightarrow 1/\sigma ^2$ and $|\phi _{2}|^2\rightarrow 1/\sigma ^4$. The second case is the standard one, $\alpha \rightarrow 1$, where we have

25 $$\begin{aligned} \phi _{1}(z)= & {} -\sigma z E_{2,2}\big ((\sigma ^2-1) z^2\big ) + E_{2,1}\big ((\sigma ^2-1) z^2\big )= -\sigma \ {\sinh \big (\sqrt{\sigma ^2-1} z\big )\over {\sqrt{\sigma ^2-1}}} + \cosh \big (\sqrt{\sigma ^2-1}\ z\big )\nonumber \\ \phi _{2}(z)= & {} i z E_{2,2}\big ((\sigma ^2-1)z^2\big )= i {\sinh \big (\sqrt{\sigma ^2-1}\ z\big )\over {\sqrt{\sigma ^2-1}}}. \end{aligned}$$

**Figure 3:**
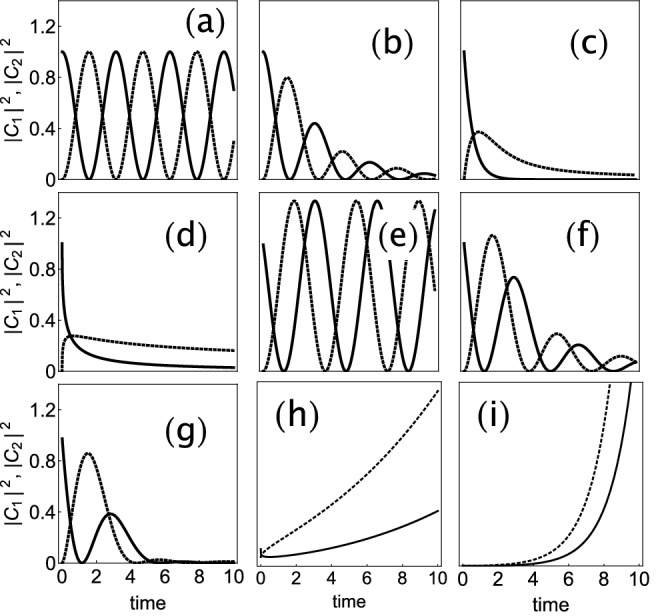
Dimer amplitudes $|\phi _{1}(z)|^2$ (continuous line) and $|\phi _{2}(z)|^2$ (dashed line) for the linear ${\mathcal{{PT}}}$ case ($\gamma =0, \sigma \ne 0$) for several fractional derivative orders and various gain/loss parameters. (**a**) $\alpha =1, \sigma =0$, (**b**) $\alpha =0.9, \sigma =0$, (**c**) $\alpha =0.5, \sigma =0$, (**d**) $\alpha =0.25, \sigma =0$, (**e**) $\alpha =1, \sigma =0.5$, (**f**) $\alpha =0.9, \sigma =0.5$, (**g**) $\alpha =0.8, \sigma =0.5$, (**h**) $\alpha =0.25, \sigma =1.1$, (**i**) $\alpha =0.8, \sigma =1.1$.

Thus, for $\sigma ^2 > 1$, we gave exponential growth, while for $\sigma ^2 < 1$ there is oscillatory behavior. This behavior at $\alpha =1$ can also be seen for other smaller $\alpha$ values, as Fig. 3 shows. The figure shows examples of time evolutions for $|\phi _{1}|^2, |\phi _{2}|^2$ for several fractional orders and several gain/loss parameter values. In general we observe that, while $\alpha$ tends to create damped oscillations, the presence of $\sigma ^2<1$ produces a degree of amplification of the oscillations and, for $\sigma ^2>1$, it gives rise to an unbounded amplification.

The asymptotic behavior of $\phi _{1}(z), \phi _{2}(z)$ depends on the behavior of the Mittag–Leffler functions $E_{\alpha ,\beta }(z)$ at large values of |*z*|. After writing $z=|z|\exp (i \phi )$, we have^32^

26 $$\begin{aligned} E_{\alpha ,\beta }(z) \approx (1/\alpha )\ Q^{1-\beta }\exp (Q) \end{aligned}$$

where $Q=z^{1/\alpha }=\exp ((1/\alpha ) \log (|z|)+ i\ \phi )$ and $|\phi /\alpha |\le \pi$ ($\phi =\sigma ^2- 1$). This implies,

27 $$\begin{aligned} \exp (Q) = \exp (|z|^{1/\alpha } \cos ((1/\alpha )\phi ))\times \exp (i\ |z|^{1/\alpha } \sin ((1/\alpha )\phi )) \end{aligned}$$

**Figure 4:**
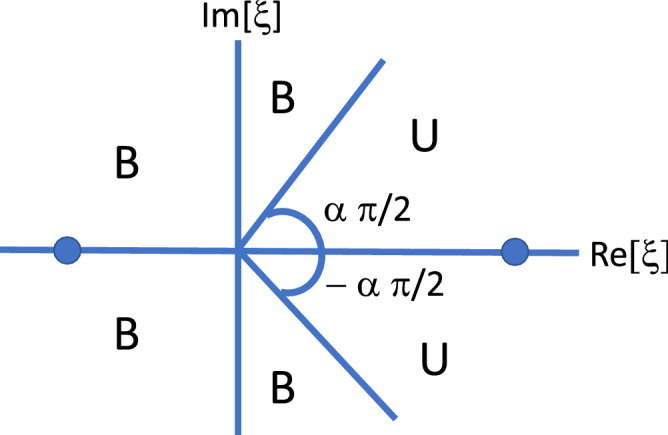
Asymptotic stability for the amplitudes $\phi _{1}(z), \phi _{2}(z)$ for the fractional ${\mathcal{{PT}}}$ dimer system (22). Here, ${\text{U }} \equiv {\text{ unbounded }}$, ${\text{B }} \equiv {\text{ bounded }}$, and $\xi =(\sigma ^2 - 1) z^{2 \alpha }$. The dots denote the position of our two phases, ${\text{ phase }}(\sigma ^2-1)=0$ for $\sigma ^2-1>0$, and ${\text{phase }}(\sigma ^2-1)=\pi$ for $\sigma ^2-1<0$.

Thus, bounded behavior in time will occur for $(\pi /2)<|\phi /\alpha |<\pi$, while unbounded behavior occurs for $0<|\phi /\alpha |<\pi /2$. In our case, $\phi ={\text{arg }}(\sigma ^2-1)=0,\pi$, implying that $\phi _{1}(z)$ and $\phi _{2}(z)$ will increase (decrease) asymptotically in time if $(\sigma ^2 - 1)$ is positive (negative). This behavior is sketched in Fig. 4.

### The nonlinear ${\mathcal{{PT}}}$ dimer

We now explore a ${\mathcal{{PT}}}$ dimer in the presence of nonlinearity, and subject to fractional evolution equations

28 $$\begin{aligned} i {d^{\alpha } \phi _{1}(z)\over {d z^{\alpha }}} + i \sigma \phi _{1}(z) + \phi _{2}(z)+ \gamma |\phi _{1}(z)|^2 \phi _{1}(z)= & {} 0\nonumber \\ i {d^{\alpha } \phi _{2}(z)\over {d z^{\alpha }}} - i \sigma \phi _{2}(z) + \phi _{1}(z) + \gamma |\phi _{2}(z)|^2 \phi _{2}(z)= & {} 0\ \ \ \ \end{aligned}$$

In the absence of ${\mathcal{{PT}}}$ symmetry ($\sigma =0$), and for order $\alpha =1$, Eq. (28) have been explored before in the literature^33–35^. For initial conditions $\phi _{1}(0)=1, \phi _{2}(0)=0$, it was shown that they lead to the phenomenon of a seltrapping transition: The existence of a critical nonlinearity parameter $\gamma _{c}=4$ below which, the long-time average of the square of the amplitudes, $\langle |\phi _{1,2}|^2 \rangle =(1/T) \int _{0}^{T} |\phi _{1,2}|^2 dz$ (with $T\gg 1$) is the same: $\langle |\phi _{1}|^2 \rangle = \langle |\phi _{2}|^2 \rangle =1/2$. At nonlinearity values above $\gamma _{c}$, $\langle |\phi _{1}|^2 \rangle$ increases past 1/2 and converges to 1 at large $\gamma$ values, while $\langle |\phi _{2}|^2 \rangle$ decreases towards zero. The trapped fraction at the initial site, $\langle |\phi _{1}|^2 \rangle$, increases abruptly as the critical nonlinearity is crossed.

In the presence of ${\mathcal{{PT}}}$ symmetry and nonlinearity both, the nonlinear dynamics of the excitation exchange and the Hamiltonian nature of the nonlinear dimer have been recently explored^36–38^. It is interesting to note that the nonlinear ${\mathcal{{PT}}}$ dimer has a Hamiltonian structure in spite of being a system containing losses and gains.

For a fractional order derivative ($0<\alpha <1$), where we take the Caputo version of the fractional derivative, and in the presence of ${\mathcal{{PT}}}$ symmetry, we resort to the Grunwald algorithm^39^ to compute the time evolution of $\phi _{1}(z), \phi _{2}(z)$ for initial conditions $\phi _{1}(0)=1, \phi _{2}(0)=0$. This approach is based on finite differences, and in our case leads to the following difference equations:

29 $$\begin{aligned} X_{n+1}= & {} \sum _{\nu =1}^{n+1} \Phi _{\nu }^{\alpha } X_{n+1-\nu } + i h \big ( Y_{n}+i \sigma X_{n} + \gamma \ |X_{n}|^2 X_{n} \big ) + r_{n+1}^{\alpha } X_{0}\nonumber \\ Y_{n+1}= & {} \sum _{\nu =1}^{n+1} \Phi _{\nu }^{\alpha } Y_{n+1-\nu } + i h \big ( X_{n}-i \sigma Y_{n} + \gamma \ |Y_{n}|^2 Y_{n} \big ) + r_{n+1}^{\alpha } Y_{0} \end{aligned}$$

where $$X\equiv \phi _{1}, Y\equiv \phi _{2}$$, and

30 $$\begin{aligned} \Phi _{\nu }^{\alpha }=(-1)^{\nu -1} \left( {\begin{array}{c}\alpha \\ \nu \end{array}}\right) r_{\nu }^{\alpha } = {\nu ^{-\alpha }\over {\Gamma (1-\alpha )}}. \end{aligned}$$

**Figure 5:**
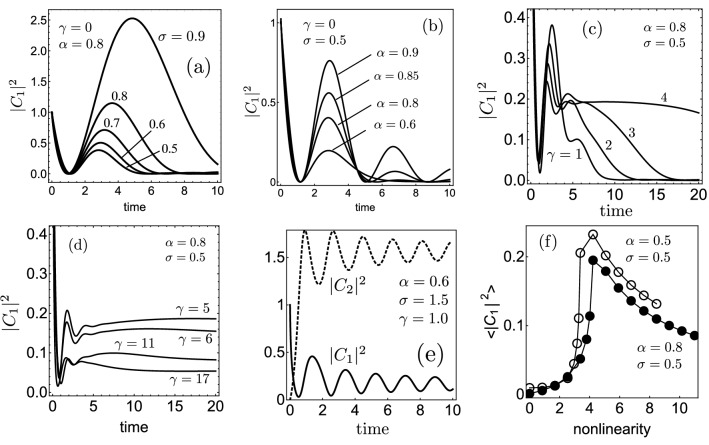
Dimer amplitude at initial site $|\phi _{1}(z)|^2$ for the nonlinear ${\mathcal{{PT}}}$ case for several fractional derivative orders $\alpha$, various gain/loss parameters $\sigma$ and different nonlinearities $\gamma$. Lower rightmost: Example of a time-averaged, trapped fraction at initial site as a function of the nonlinearity parameter ($\sigma =0.5, \alpha =0.8$).

Numerical results are shown in Fig. 5. In panel (a) we show the behavior of $|\phi _{1}(z)|^2$ in the linear limit ($\gamma =0$), for a fixed $\alpha$ and several different gain/loss parameter values. We see that the effect of increasing $\sigma$ is to augment the amplitude and decrease the frequency of the oscillation. When $\sigma$ approaches 1, the amplitude grows unbounded and the oscillation stops. In panel (b) we take $\gamma =0$ as before, with a fixed gain/loss $\sigma$ and for several order $\alpha$ values. As we noticed before, the presence of $0<\alpha <1$ induces a decreasing oscillation behavior in $|\phi _{1}(z)|^2$. If we reduce now the value of $\alpha$, we see a further decrease of the oscillation amplitude, with little effect on the frequency. We now move to the nonlinear case. In panels (c, d) we show the behavior of $|\phi _{1}(z)|^2$ in time for fixed $\alpha , \sigma$ parameters and for several $\gamma$ values. Roughly speaking, what we observe here is that there seems to exist a special nonlinearity value below which the curves decrease steadily to zero at long times, and above which they approach a constant nonzero value in time. To help understand this, we show in Fig. 5e $\langle |\phi _{1}|^2\rangle =(1/T)\int _{0}^{T}|\phi _{1}|^2 dz$ $(T\gg 1)$, the time-averaged fraction remaining at the initial site vs the nonlinearity strength. As soon as the nonlinearity increases from zero there is a finite amount of trapping at the initial site that increases monotonically with nonlinearity. As the nonlinearity parameter reaches a critical value $\gamma _{c}$ whose precise value depends on $\alpha$, there is an abrupt increase in $\langle |\phi _{1}|^2\rangle$ signaling a seltrapping transition, like in the standard ($\alpha =1$) nonlinear dimer. What is interesting though, is that if we continue increasing the nonlinearity past the critical point, the trapped fraction begins to decrease instead of increasing towards unity as in the standard case. This fragility of the trapping could perhaps be a manifestation of the tendency of $\alpha$ to decrease the amplitude of oscillations in general. Thus, what we are seeing here is the interplay of two opposing tendencies: Trapping by nonlinearity and amplitude decay by $\alpha$.

## Conclusions

We have examined the excitation dynamics in a nonlinear ${\mathcal{{PT}}}$ dimer when the evolution equations are ruled by a fractional-order time derivative, instead of the usual first-order. In the absence of nonlinearity we solved the fractional ${\mathcal{{PT}}}$ dimer equations in closed form, using the formalism of Laplace transform, obtaining a solution in terms of Mittag–Leffler functions.

The system is admittedly a complex one since it combines several behaviors stemming from fractionality, gain/loss and nonlinearity. The general solution shows that, in the absence of gain/loss and nonlinearity, the effect of the fractional derivative alone is to induce a damping in the oscillatory exchange between the two sites. When gain/loss effects are added, we observed two regimes: For gain/loss parameter smaller than a certain threshold, we observe an amplification of the oscillatory amplitudes. The $\alpha \rightarrow 0$ case shows that, for gain/loss below threshold, the dynamics is sinusoidal, while above threshold it leads to an exponential growth.

Finally, when nonlinearity is added to the picture, and for a gain/loss below threshold, we observe selftrapping at long times at the initial site, that increases steadily as the nonlinearity reaches a critical value. Above this nonlinear threshold, the trapped fraction at the initial site decreases monotonically as nonlinearity is increased further. This is in marked contrast with the standard case where an increase in selftrapping produces a monotonic increase in selftrapping. When the gain/loss parameter is above threshold, the unbounded behavior can be arrested by the selftrapping tendency of the nonlinearity giving rise to a non-zero saturation of the amplitudes as a function of time.
